# Risk Factors for Seroconversion by *Leishmania infantum* in a Cohort of Dogs from an Endemic Area of Brazil

**DOI:** 10.1371/journal.pone.0071833

**Published:** 2013-08-22

**Authors:** Wendel Coura-Vital, Alexandre Barbosa Reis, Maria Arlene Fausto, Gleisiane Gomes de Almeida Leal, Marcos José Marques, Vanja Maria Veloso, Mariângela Carneiro

**Affiliations:** 1 Pós-Graduação em Infectologia e Medicina Tropical, Faculdade de Medicina, Universidade Federal de Minas Gerais, Belo Horizonte, Minas Gerais, Brazil; 2 Núcleo de Pesquisas em Ciências Biológicas, Instituto de Ciências Exatas e Biológicas, Universidade Federal de Ouro Preto, Ouro Preto, Minas Gerais, Brazil; 3 Departamento de Parasitologia, Instituto de Ciências Biológicas, Universidade Federal de Minas Gerais, Belo Horizonte, Minas Gerais, Brazil; 4 Departamento de Alimentos, Escola de Nutrição, Universidade Federal de Ouro Preto, Ouro Preto, Minas Gerais, Brazil; 5 Departamento de Ciências Biológicas, Universidade Federal de Alfenas, Alfenas, Minas Gerais, Brazil; 6 Departamento de Farmácia, Escola de Farmácia, Universidade Federal de Ouro Preto, Ouro Preto, Minas Gerais, Brazil; Technion-Israel Institute of Technology Haifa 32000 Israel, Israel

## Abstract

Visceral leishmaniasis (VL) has recently emerged in various urban and peri-urban areas of Brazil and other countries. Understanding the urbanization of VL requires identification of risk factors associated with human and canine infection. To determine the predictors of risk for canine VL, a survey was conducted of 1,443 dogs, from which a cohort was selected (*n* = 455) and evaluated for approximately 26 months. Serology was conducted with two enzyme-linked immunosorbent assays (ELISA): one conducted in the Laboratory of Zoonosis of the Belo Horizonte Health Department (LZOON) and the other in the Laboratory of Immunopathology of the Federal University of Ouro Preto (LIMP). A molecular diagnostic method (PCR–restriction fragment length polymorphism) and a structured questionnaire were also used. To identify the factors associated with seroconversion, two time-dependent Cox regression models were performed with different sensitivities (model 1, seroconversion by ELISA/LZOON; model 2, seroconversion by ELISA/LIMP). The overall incidences of seroconversion were 6.5/1000 dogs-months and 11.2/1000 dogs-months for ELISA/LZOON and ELISA/LIMP, respectively. Increased risk of seroconversion was associated with short fur (model 1: hazard ratio [HR] 1.9), the presence of dry leaves (model 1: HR 2.8) or manure (model 1: HR 3.5) in the backyard, dogs sleeping predominantly in the backyard (model 2: HR 2.1), the presence of symptoms (model 2: HR 2.0), and positive molecular results during follow-up (model 2: HR 1.5). Decreased risk was associated with insecticide spraying in the house (model 2: HR 0.5). These results indicate that more-vulnerable domiciles, certain dog behaviors, lack of vector control measures, and positive molecular results were associated with the occurrence of canine VL. Furthermore, it is important to emphasize that PCR-positive dogs should be monitored, owing to the possibility of seroconversion. Identifying risk factors for seroconversion in dogs is crucial for developing adequate strategies for VL prevention and control.

## Introduction

Visceral leishmaniasis (VL) is a neglected disease with an estimated incidence of 500,000 new cases and 59,000 deaths annually [1]. In South America and Europe, it is caused by the protozoan parasite *Leishmania infantum* and is transmitted by phlebotomine sand fly vectors [2].

Since the early 1980s, VL has been spreading to the urban centers of northern Brazil and, more recently, to southern and western regions [3]. To reduce morbidity and case-fatality rates, the Brazilian Ministry of Health, through the Visceral Leishmaniasis Control and Surveillance Program (VLCSP), has instituted specific measures, including early diagnosis and treatment of human cases, vector control with insecticides, serological screening and subsequent culling of infected dogs, and health education [4]. At present, Brazil is the only country where seropositive dogs are systematically removed [5]. Despite these measures, the number of reported human cases increased from 1,944 in 1990 to 3,894 in 2011 [6]–[7].

Understanding the expansion and urbanization of VL requires identification of the risk factors associated with human and canine infection. Little is known about the risk factors for canine infection. Cross-sectional serological surveys have suggested that susceptibility to infection is associated with dog size, fur length, age, and living outdoors [8]–. However, few studies have evaluated risk factors using a cohort study [11], which is the most appropriate observational design to establish causal inference. A cross-sectional study conducted demonstrated that factors associated with early *L. infantum* infection were the lower socioeconomic status of the owner, dog behavior, the owner’s knowledge about the vector, and the care given to the dogs [12].

In Brazil, the VLCSP has recently used the Dual Path Platform (Bio-Manguinhos/Fiocruz, Rio de Janeiro, Brazil) to screen dogs and enzyme-linked immunosorbent assay (ELISA) to confirm positive results [13]. Among the molecular screening methods, polymerase chain reaction (PCR) can detect infection before seroconversion [14]–[15], but it is important to follow up PCR-positive dogs to monitor seroconversion during the course of *L. infantum* infection.

Herein, we report the results of a concurrent cohort study that was designed to estimate the incidence rate of seroconversion in dogs over time and to identify the risk factors associated with seroconversion, including the domiciliary and peridomiciliary environment, the socioeconomic status of the owners, the care given to the animals, and the animals’ characteristics and behavior. The study was conducted in Belo Horizonte, the capital of Minas Gerais, which is located in southeastern Brazil and has one of the highest incidences of human VL in the country, varying from 1.2/100,000 (in 1998) to 7.2/100,000 inhabitants (in 2008) [3], [16]. Furthermore, the proportion of seropositive dogs has ranged from 7% to 10% in the last few years [3], [12]. The cohort design was conducted using ELISAs and PCR as the diagnostic methods; in addition, several other variables were measured at approximately 6-month intervals over the course of 26 months. Dynamic survival models of the extended Cox model were used to capture the time variation of the relationships between the variables.

## Methods

### Ethical Statement

The study was approved by the Committee of Ethics in Animal Experimentation of the Federal University of Ouro Preto (protocol no. 083/2007), of the Federal University of Minas Gerais (protocol no. 020/2007), and of the city council of Belo Horizonte (protocol no. 001/2008). All procedures followed guidelines set forth by the Brazilian Animal Experimental College (federal law number 11794). Dog owners were informed of the research objectives and were required to sign an informed consent form before sample and data collection.

### Initial Survey

The rationale and organization of the study and the methods of data collection have been described elsewhere [12]. Briefly, a cross-sectional study was conducted in 2008 in the northwest sanitary district (36,874 km^2^) of Belo Horizonte. According to a census conducted by the Brazilian Institute of Geography and Statistics, this area’s population was 331,362 in 2010. The canine population comprised 20,883 animals, according to the Zoonosis Control Management of the northwest sanitary district. At the time of the study, the canine VL (CVL)–positive rates in Belo Horizonte and its northwest sanitary district were 7.6% and 7.8%, respectively [17]. Using an expected CVL positivity in the study area of between 5% and 10%, the 95% confidence interval (CI), and an estimated precision of 1.5%, it was estimated that the appropriate sample size for the study was approximately 1,500 animals, which would permit identification of enough PCR-positive dogs among seronegative animals for the cohort. This estimate accounted for the greater sensitivity of PCR and for loss to follow-up. The field work was done in close collaboration with the Municipality Health Service, and the data were collected during the canine survey census conducted by health agents as part of VLCSP’s routine. The study area was selected for convenience and because a canine survey was starting in the area in 2008 [12]. Households visited by the VLCSP in an area that comprised 37 census tracts (according to the Brazilian Institute of Geography and Statistics [18]) were included in the study.

### Follow-up Studies

For the cohort study, sample size was calculated using the following parameters: seroconversion rate of 10%, power of 80%, 95% CI, and relative risk of 2.0. The initial plan was to follow up 400 seronegative dogs, 200 in each groups based on PCR results (seronegative/PCR+, seronegative/PCR–).

**Figure 1:**
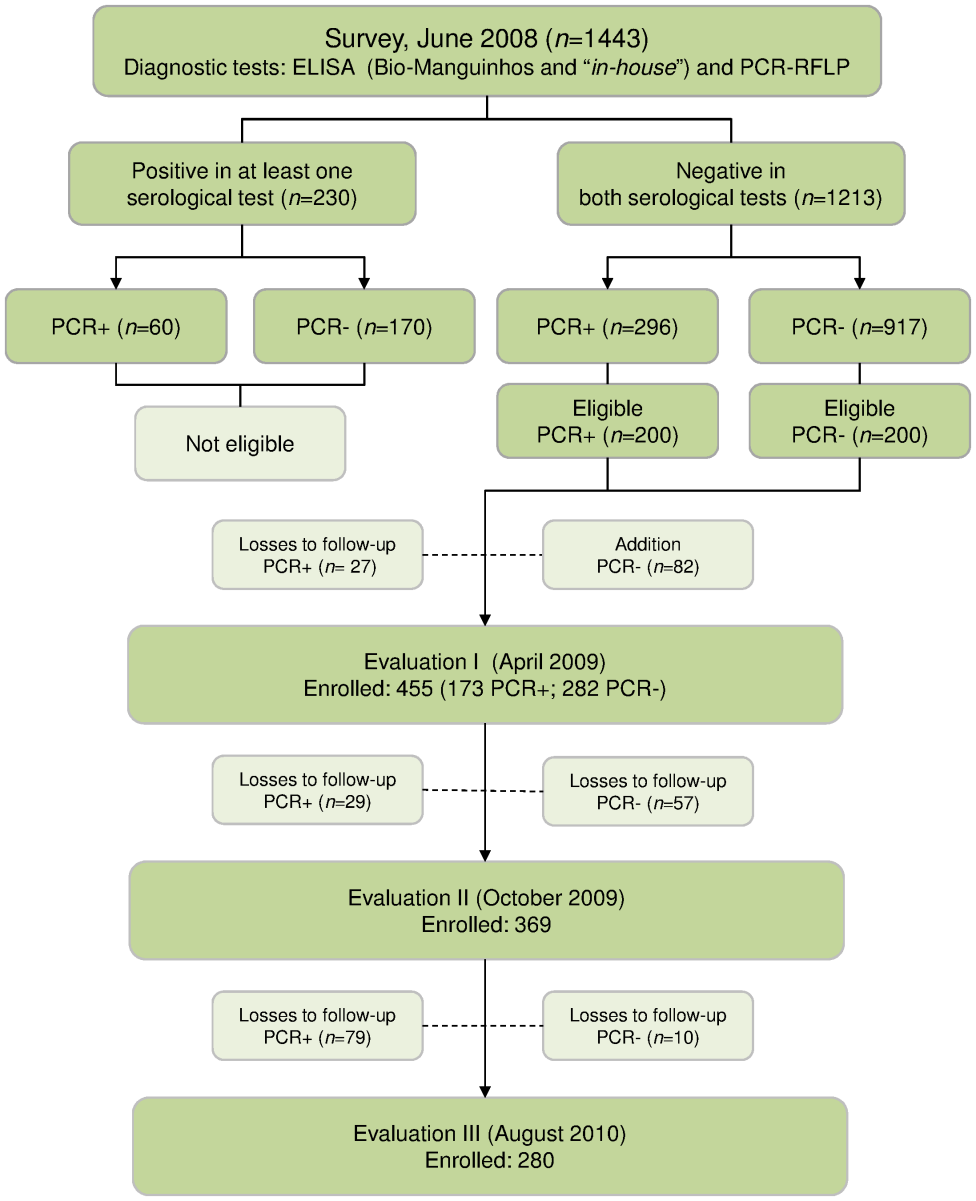
Baseline survey; evaluations I, II, and III; and losses to follow-up, Belo Horizonte, Minas Gerais.

The first follow-up study was initiated 10 months after the baseline survey (April 2009, designated evaluation I) during the VLCSP canine survey census, and a total of 455 dogs were enrolled. Households with seronegative/PCR– dogs were selected by proximity to households with seronegative/PCR+ dogs. The canines were selected from 333 owners, who were interviewed with a precoded questionnaire to identify risk factors related to seroconversion. All dogs were clinically examined, and blood was collected by venipuncture. Evaluation II was conducted 16 months after the baseline survey (October 2009), and 369 dogs were included. Evaluation III was carried out 26 months after the baseline survey (August 2010), and 280 dogs were tested (Figure 1). All the dogs included in evaluations II and III were subjected to the same procedures used for evaluation I.

The reasons for losses during follow-up were euthanasia (due to seroconversion), death, change of address, household closed, refusal, and dog escape. The closed houses were visited 3 times before the dogs were considered lost to follow-up. To test trends in losses during the cohort study, the variables sex, size, and fur length were compared between the dogs included and not included in each evaluation phase and no differences were observed.

### Data Collection

The owners of the study animals were interviewed by a trained research team using a previously tested structured questionnaire that sought information regarding (i) knowledge about the disease (i.e., form of transmission and clinical signs of human VL [HVL]); (ii) knowledge about the vector (characteristics and presence in the domicile and peridomicile); (iii) knowledge about the host (epidemiological importance of the host, clinical signs of leishmaniasis, and care of the dog); (iv) socioeconomic characteristics of the owner (per capita/family income and schooling); (v) characteristics of the domicile, annexes, and surroundings (i.e., structure of roof, floor, and walls; number of rooms, including bedrooms; number of residents; presence of trees [particularly banana trees], rubble, manure, exposed garbage, dry leaves, and vegetable garden); (vi) method of garbage disposal (collected, burned, or buried); and (vii) presence of other animals (birds, cats, or cattle). Knowledge about the disease was validated according to self-reporting of the main symptoms of CVL and HVL. Vector recognition was acknowledged by self-reporting and validated by showing different Diptera species samples (*Lutzomyia longipalpis* and *Aedes aegypti*) to the participants. The following information was collected for each dog: age, sex, size, fur length, breed, behavior (where the dog habitually slept and spent most of its time, e.g., street, residence, or backyard), dog care, clinical examinations, past history of vaccination, and serological exams previous to leishmaniasis. Age was estimated according to owner’s reports. The dogs were also categorized as asymptomatic (with no signs suggestive of CVL) or symptomatic (with characteristic clinical signs of CVL, such as opaque bristles, severe loss of weight, onychogryphosis, cutaneous lesions, apathy, and keratoconjunctivitis).

### Collection of Blood Samples

A sample of peripheral blood (5 mL) was collected by puncture of the brachiocephalic vein, and an aliquot was transferred to a glass vial containing sufficient anticoagulant (ethylenediaminetetraacetic acid [EDTA]) to achieve a final concentration of 1 mg/mL. The blood sample was centrifuged (1500–1800 × *g* for 20 min), and the buffy coat fraction containing the leukocytes was removed, resuspended 1∶1 (v/v) in 10 mM Tris-HCl buffer (supplemented with 1 mM EDTA), and stored at –70°C until required for PCR–restriction fragment length polymorphism (RFLP). The remainder of the blood sample was transferred to two separate filter papers for subsequent ELISA analysis. The serological and molecular tests were performed approximately 1 month after blood collection.

### ELISA Protocols

Each eluate from blood dried on filter paper was tested by means of 2 ELISA protocols. The first protocol used an EIE-LVC kit (Bio-Manguinhos/Fiocruz, Rio de Janeiro, Brazil), which employs a soluble antigen from promastigote forms of *L. major*-like (ELISA-*L. major*-like). This serological analysis was performed in the Laboratory of Zoonosis of the Belo Horizonte Health Department (LZOON) according to the kit manufacturer’s instructions. Sensitivity and specificity were 98% and 95%, respectively [19]. The second protocol was performed in parallel by using soluble *L. infantum* (MHOM/BR/1070/BH46) antigen and was carried out in the Laboratory of Immunopathology of the Federal University of Ouro Preto (LIMP) as described by Coura-Vital et al. [12]. Sensitivity and specificity were 100% and 88%, respectively [20]. The cut-off value was established as the mean absorbance value +2 standard deviations (SDs) from 20 eluates from blood of uninfected dogs collected from a non-endemic area and dried on filter paper.

### Molecular Method (PCR-RFLP)

DNA was extracted from the buffy coat fractions by means of Wizard Genomic DNA purification kits (Promega Corporation, Madison, WI, USA) according to the manufacturer’s instructions. The primers used to amplify the conserved region of the *Leishmania* kDNA minicircle were as follows: forward, 5′-GGG (G/T)AG GGG CGT TCT (G/C)CG AA-3′; reverse, 5′-(G/C)(G/C)(G/C) (A/T)CT AT(A/T) TTA CAC CAA CCC C-3′ [21]. A single PCR product of 120 bp was generated [22]. The reaction was performed as described by Coura-Vital et al. [12].

PCR amplicons (5 µL) were digested for 3 h at 37°C in 1 U of *Hae*III (Invitrogen, São Paulo, Brazil) in 1X buffer (10 mM Tris-HCl, 10 mM MgCl_2_ [pH 7.5]) and enough Milli-Q water to bring the final volume to 15.0 µL/well (MicroAmp Fast Optical 96-Well Reaction Plate, Applied Biosystems) [23]. Restriction fragments, together with a 25-bp DNA ladder (Invitrogen), were subjected to electrophoresis in 10% polyacrylamide gels at 40 mA in 89 mM Tris base (pH 8.0), 89 mM boric acid, and 2 mM EDTA. Bands were detected by silver staining, and the patterns were compared with those obtained with DNA from *L*. (*L.*) *amazonensis* (strain MHOM/BR/1973/M2269), *L*. (*Viannia*) *braziliensis* (strain MHOM/BR/1975/M2903), and *L.* (*L.*) *infantum* (strain MHOM/BR/1972/BH46) from the DNA reference library at LIMP. Sensitivity and specificity were 91% and 61%, respectively [23].

### Statistical Analysis

Databases were generated using EpiData (version 3.2, EpiData Association, Odense, Denmark) by double entry of the results, and the data were subsequently corrected, compared, and analyzed with Stata software (version 11.0, Stata Corporation, College Station, TX, USA).

A dynamic survival model or time-dependent Cox regression model was used to evaluate the risk factors associated with seroconversion. This model was used due to time-dependent variables that changed as a function of follow-up time, such as age, PCR-RFLP, and symptomatology [24]. The other variables in the model did not vary with time.

Two models were constructed on the basis of a different measurement of seroconversion: model 1, seroconversion measured by ELISA/LZOON; model 2, seroconversion measured by ELISA/LIMP.

With regard to censorship, the moment of loss was placed halfway between the last collection and the time of accompaniment. Variables that were statistically significant but exhibited colinearity were excluded from the multivariate analysis, and categorical variables were transformed into dummy variables.

We performed the univariate analysis using time-dependent Cox regression models to examine the associations between each variable and time to seroconversion. A multivariable adjusted model was fitted with the variables that were statistically significant at *p*<0.25 in univariate analyses. A step-by-step backward selection procedure was used to select the variables and to produce the final multivariate regression models. Only adjusted variables showing a significant association (*p*<0.05) with the occurrence of seroconversion from CVL remained in the final models. The strength of association was determined by hazard ratios (HRs) at a 95% CI. The Schoenfeld test was performed to test the proportional hazards assumption.

## Results

### Follow-up Phases

#### Evaluation I

The original plan was to follow 400 dogs (200 in each group); however, 27 seronegative/PCR+ dogs were lost to follow-up, and 82 seronegative/PCR– dogs were added, for a total of 455 dogs enrolled.

#### Evaluation II

In this phase, 369 dogs were analyzed; 29 and 57 dogs were lost to follow-up among the PCR-positive and PCR-negative groups, respectively.

#### Evaluation III

A total of 280 dogs were included; 89 dogs (79 PCR+, 10 PCR–) were lost to follow-up (Figure 1).

### Characteristics of the Dogs

The characteristics of the 455 dogs included in follow-up were similar to those of the dogs evaluated at the baseline survey. Female (51.6%), medium-sized (52.7%), and short-haired (57.8%) animals predominated. The mean age was 53.7 months (SD 42.1), and the median (interquartile range, IQR) was 48 months (24; 72), and the majority of the animals (43.5%) were between 24 and 84 months old. Generally, the animals lived and slept in the backyard (86.7% and 82.9%, respectively), rather than inside the residence. Most of the dogs (51.9%) had received a check-up by a veterinarian (Table 1). Of the animals included in the follow-up 96.5% were asymptomatic.

**Table 1:** Characteristics of dogs in the cohort study, Brazil 2010.

Variable	PCR		Total (%)
	No. positive dogs (%)	No. negative dogs (%)	
Sex			
Female	94 (51.6)	141 (50.0)	235 (51.6)
Male	79 (48.4)	141 (50.0)	220 (48.4)
Size			
Small	41 (23.7)	81 (28.7)	122 (26.8)
Medium	99 (57.2)	141 (50.0)	240 (52.7)
Big	33 (19.1)	60 (21.3)	93 (20.5)
Fur length			
Short	102 (59.0)	161 (57.1)	263 (57.8)
Long	71 (41.0)	121 (42.9)	192 (42.2)
Age			
≤24 months	59 (34.1)	97 (34.4)	156 (34.3)
>24 and ≤84 months	75 (43.4)	123 (43.6)	198 (43.5)
>84 months	39 (22.5)	62 (22.0)	101 (22.2)
Dog staying predominantly in the backyard			
Yes	139 (85.2)	247 (87.6)	386 (86.7)
No	24 (14.8)	35 (12.4)	59 (13.3)
Sleeping place			
In the backyard	133 (81.5)	236 (83.7)	369 (82.9)
Inside the house	30 (18.5)	46 (16.3)	76 (17.1)
Veterinary check-ups			
Yes	90 (55.5)	141 (50.0)	231 (51.9)
No	73 (44.5)	141 (50.0)	214 (48.1)
Vaccine for leishmaniasis			
Yes	7 (4.0)	11 (4.3)	18 (4.2)
No	167 (96.0)	244 (95.7)	411 (95.8)

### Housing and Environmental Conditions

A total of 333 households were selected. They had a mean (SD) of 1.38 (0.82) dogs per household (1–7 dogs/house) and median of 1 (IQR 1; 2). The majority of the dwellings (208; 62.5%) were detached houses; 310 (93.1%) had plastered walls, 262 (78.7%) had floors constructed with ceramics/wood, 321 (96.4%) were served by main sewage, and 272 (81.7%) were sprayed with insecticide. Garbage was collected 3 or more times per week from 294 (88.3%) residences. The mean (SD) numbers of rooms and bedrooms per house were 7.1 (2.8) and 2.6 (1.0), respectively. Each dwelling had an average of 1.7 (0.8) residents. Most houses (267; 80.2%) had a backyard; 33 (10.0%) and 7 (2.1%) had dry leaves and manure in the backyard, respectively (data not shown).

### Risk Factors Associated with Seroconversion

The risk for seroconversion was evaluated on the basis of ELISA results obtained with two different antigens. According to LZOON, 65 failure events were observed within the cohort, with overall incidence rate of 6.5/1000 dogs-months (95% CI 5.1–8.2). At LIMP, 109 failure events were observed, with an incidence rate of 11.2/1000 dogs-months (95% CI 9.3–13.5) (Table 2). The dogs reagents for serology performed at LZOON were euthanized by the Centre for Zoonosis Control of Belo Horizonte, as recommended by the Brazilian Ministry of Health [4].

**Table 2:** Dog-months of follow-up, failure events (seroconversion), and incidence rates with 95% CIs, Brazil 2010.

	ELISA seroconversion			
Follow-up	LZOON		LIMP	
	Failure events	Incidence rate^d^ (95% CI)	Failure events	Incidence rate^d^ (95% CI)
Evaluation I^(a)^	34	6.3 (4.5–8.8)	21	2.0 (1.1–3.7)
Evaluation II^(b)^	12	5.4 (3.1–9.6)	29	13.5 (9.4–19.5)
Evaluation III^(c)^	19	7.8 (5.0–12.2)	69	31.4 (24.8–39.8)
Total	65	6.5 (5.1–8.2)	109	11.2 (9.3–13.5)

a, b, c 10, 16, and 26 months after baseline, respectively;

d Incidence rate/1000 dogs-months. Abbreviations: LZOON, Laboratory of Zoonosis of the Belo Horizonte Health Department; LIMP, Laboratory of Immunopathology of the Federal University of Ouro Preto; CI, confidence interval.

The results of preliminary selection of the variables from the univariate analysis (*p*>0.25) are shown in Tables 3 and 4. The risks factors according to the time-dependent Cox regression model are shown in Table 5. Incidence of seroconversion detected by ELISA/LZOON was associated with short fur (HR 1.9; 95% CI 1.1–3.4) and with the presence of dry leaves (HR 2.8; 95% CI 1.6–5.0) and manure (HR 3.5; 95% CI 1.3–9.7) in the backyard. Incidence of seroconversion detected by ELISA/LIMP was associated with sleeping predominantly in the backyard (HR 2.1; 95% CI 1.1–4.1), presence of the symptoms during follow-up (HR 2.0; 95% CI 1.1–3.9), and positive molecular results during follow-up (HR 1.5; 95% CI 1.4–3.9). Moreover, insecticide spraying in the house (HR 0.5; 95% CI 0.3–0.8) reduced the seroconversion incidence.

**Table 3:** Univariate analysis (*p*<0.25) according to the characteristics of the dogs (*n* = 455), Brazil 2010.

	Event (ELISA seroconversion)				
Variable	LZOON		LIMP		
	HR (95% CI)	*p*	HR (95% CI)		*p*
**Sex**					
Male vs. female	1.0 (0.6–1.6)	0.86	1.2 (0.8–1.8)		0.24
**Fur length**					
Short vs. long	2.5 (1.2–3.6)	0.01	1.2 (0.8–1.8)		0.24
**Origin of the animal**					
Other district vs. district of residence	0.6 (0.34–0.97)	0.04	0.8 (0.5–1.2)		0.27
**Dog staying predominantly in the backyard**					
Yes vs. no	3.2 (0.9–10.0)	0.05	1.5 (0.8–3.0)		0.23
**Sleeping place**					
In the backyard vs. inside the house	3.1 (1.1–8.5)	0.03	1.6 (0.9–3.0)		0.12
**Veterinary check ups**					
Yes vs. no	0.6 (0.4–1.1)	0.08	0.9 (0.6–1.3)		0.49
**Symptomatic**					
Yes vs. no	2.2 (1.0–4.8)	0.05	1.7 (0.9–3.4)		0.10
**PCR**					
Positive vs. negative	1.0 (0.6–1.8)	0.99	2.4 (1.4–3.9)		0.14

Abbreviations: LZOON, Laboratory of Zoonosis of the Belo Horizonte Health Department; LIMP, Laboratory of Immunopathology of the Federal University of Ouro Preto; HR, hazard ratio; CI, confidence interval.

**Table 4:** Univariate analysis (*p*<0.25) according to the understanding of the disease and environmental conditions, Brazil 2010.

	Event (ELISA seroconversion)			
Variables	LZOON		LIMP	
	HR (95% CI)	*p*	HR (95% CI)	*p*
**Understanding the disease**				
Regarding the disease				
Yes vs. no	2.4 (0.3–17.2)	0.39	4.5 (1.1–18.3)	0.04
Regarding the transmission				
Yes vs. no	0.6 (0.4–1.1)	0.08	0.7 (0.5–1.0)	0.06
Regarding symptoms in the dog				
Yes vs. no	0.8 (0.5–1.3)	0.33	0.7 (0.5–1.0)	0.07
Regarding the vector				
Yes vs. no	0.9 (0.5–1.8)	0.79	0.6 (0.4–1.2)	0.06
Seen the vector in the henhouse				
Yes vs. no	2.1 (0.5–8.8)	0.29	4.0 (0.6–28.9)	0.16
**Environmental conditions**				
Insecticide-sprayed house				
No vs. yes	1.5 (0.6–3.9)	0.35	0.5 (0.3–0.9)	0.05
Floor construction				
Other vs. ceramics/wood	1.7 (1.0–2.9)	0.06	1.3 (0.8–2.0)	0.24
Presence of a backyard				
Yes vs. no	1.8 (0.8–4.2)	0.17	1.1 (0.6–1.8)	0.84
Dry leaves in the backyard				
Yes vs. no	3.0 (1.7–5.3)	0.00	1.4 (0.8–2.4)	0.24
Manure in the backyard				
Yes vs. no	4.0 (1.4–11.1)	0.05	3.1 (1.1–8.4)	0.03

Abbreviations: LZOON, Laboratory of Zoonosis of the Belo Horizonte Health Department; LIMP, Laboratory of Immunopathology of the Federal University of Ouro Preto; HR, hazard ratio; CI, confidence interval.

**Table 5:** Risk factors for canine visceral leishmaniasis according to the time-dependent Cox regression model, Brazil 2010.

	Final model (ELISA seroconversion)	
Variable	LZOON	LIMP
	Adjusted HR (95% CI)	Adjusted HR (95% CI)
**Fur length**		
Short vs. long	1.9 (1.1–3.4)	
**Sleeping place**		
In the backyard vs. inside the house		2.1 (1.1–4.1)
**Dry leaves in the backyard**		
Yes vs. no	2.8 (1.6–5.0)	
**Manure in the backyard**		
Yes vs. no	3.5 (1.3–9.7)	
**Symptoms during study**		
Yes vs. no		2.0 (1.1–3.9)
**Insecticide-sprayed house**		
Yes vs. no		0.5 (0.3–0.8)
**PCR**		
Positive vs. negative		1.5 (1.4–3.9)

Abbreviations: LZOON, Laboratory of Zoonosis of the Belo Horizonte Health Department; LIMP, Laboratory of Immunopathology of the Federal University of Ouro Preto; HR, hazard ratio; CI, confidence interval.

## Discussion

Three important features must be highlighted: (i) the epidemiological design used (i.e., concurrent cohort) to evaluate seroconversion, (ii) the large number of household dogs evaluated in an urban endemic area, and (iii) the characteristic of the dogs selected to compose the cohort (seronegative/PCR+ and seronegative/PCR– dogs). The present investigation showed that certain dog characteristics and behaviors, peridomicile conditions, and lack of insecticide spraying in the house were positively associated with seroconversion by *L. infantum* in dogs. Furthermore, our results show for the first time the importance of a PCR-positive test as a factor associated with seroconversion for *L. infantum*; the molecular test can detect infection before seroconversion, suggesting that PCR+ dogs should be monitored throughout serological test. These results are relevant because they improve our understanding of the transmission of CVL in large cities such as Belo Horizonte, where VLCSP guidelines have been followed since 1993 but the incidence of VL has not been reduced [3].

The incidence of canine infection is an important epidemiologic parameter to consider in prioritizing target control areas, because seropositive dogs (including asymptomatic dogs) can infect phlebotomine sand flies [25]–[26]. In the present study, the incidence of seroconversion by ELISA methods at LZOON and LIMP was evaluated. An increased incidence of seroconversion was observed during follow-up by ELISA/LIMP, but the ELISA/LZOON results did not follow the same pattern. A possible explanation is that the ELISA/LZOON kit currently distributed for public use in Brazil uses the *L. major*-like antigen [19], and the accuracy of this test has been questioned [27]. In contrast, ELISA/LIMP uses an antigen (soluble *L. infantum* antigen) that offers greater accuracy for eluates from blood collected on filter paper [20]. Another factor that may have influenced our results is the large number of asymptomatic dogs at follow-up. These animals usually presented low serological titers [28]–[29], causing borderline and discordant results in different diagnostic tests.

In a cohort study that evaluated diagnostic methods in asymptomatic dogs, Otranto et al. [30] observed that there is no gold standard for the detection of *Leishmania* infections in these animals. These authors suggested that more than one test should be used for the diagnosis of CVL in endemic areas, because some fraction of infected dogs may never seroconvert [31]. It is noteworthy that, according to Courtenay et al. [32], infected dogs (as evaluated by PCR or culture) are not infectious before seroconversion; thus it is important to evaluate the incidence of seroconversion in endemic areas. Our results demonstrate that Belo Horizonte is an area of active CVL transmission and of higher HVL risk, because infection in dogs generally precedes human cases.

One control measure taken by the VLCSP that has had little impact is the elimination of the canine reservoir, and this failure has been ascribed to delays in detecting and eliminating infected dogs, the tendency to replace infected dogs with susceptible puppies, and the low sensitivity of the available serological methods [33]–[34]. Furthermore, the effectiveness euthanasia of seropositive dog in controlling VL is widely questioned [35]–[36]. There is no scientific evidence indicating that dog culling reduces the incidence of the disease in humans [5]. According to Quinnell and Courtenay [37], the efficacy of dog culling would be increased if only those dogs that were infectious could be identified and killed. However, at present there are no diagnostic methods that reliably distinguish between infectious and non-infectious dogs. In view of this problem, one strategy that might help control the disease would be to improve our understanding of the risk factors associated with CVL.

It was observed that in symptomatic dogs, short fur and sleeping in the backyard were risk factors for seroconversion; the risk for these dogs was approximately twice that for dogs without these characteristics. However, the lower limits of the CIs for these variables were close to 1, indicating that the importance of these risk factor is arguable. Nevertheless, we retained these variables in the final models, to allow for better adjustment of the data. The association between the presence of symptoms and seroconversion was due to the higher levels of anti-*Leishmania* IgG in symptomatic dogs [29], [38]–[39]. The apparent protection conferred by long fur may have been due to interference with the sand fly’s ability to bite [11]. The activity of sand flies are crepuscular or nocturnal and they bite the most immediately after sunset; biting rates are 7 times as high outdoors as indoors [2], [40]. We observed that dogs that usually slept in the backyard were 2 times as likely to undergo seroconversion as those that slept inside the house. A similar association between seroconversion and sleeping outside was found in a cross-sectional study in which the prevalence of infection was evaluated by means of PCR-RFLP [12].

Current guidelines suggest the use of quantitative serology followed by PCR for diagnosis of CVL [41], and this combination allows the detection of most infected dogs [42]. Courtenay et al. [32] reported that infectiousness in infected dogs appears to correlate with anti-*Leishmania* antibody levels, PCR positivity, and clinical score. A relevant outcome of the present follow-up was recognition of the importance of monitoring seronegative/PCR+ dogs. Dogs that were PCR positive had approximately twice the risk of seroconversion as PCR-negative dogs. This result supports the hypothesis that seronegative/PCR+ animals have had previous contact with the parasite and may develop the disease [14]–[15].

The presence of manure and/or dry leaves in the backyard was associated with approximately 3 times the risk of seroconversion. The need for organic matter as a food source for sand fly larvae [2], [43] likely plays an important role in the survival of the species in peridomiciliary environments. In another study, the risk of canine infection was higher in houses with abundant nearby vegetation [44].

Among the control measures evaluated in this study, the use of insecticide spraying during the year prior to the study was significantly associated with reduced canine seroconversion. Dogs living in homes that were sprayed with pyrethroid insecticides had half the risk of seroconversion compared to dogs in unsprayed homes. Because insecticide spraying is typically limited to the intra- or peridomiciliary environment, spraying aims to reduce biting rates within and around houses [37]. However, controlled studies evaluating the effect of household spraying on the incidences of CVL and HVL are needed [45]. Some other preventive measures could be adopted to reduce the risk of CVL, including keeping dogs in closed kennels or indoors during periods of intense vector activity. The use of insecticide-impregnated collars also protects against CVL [46]–[48], but the cost of such collars is prohibitively high for most of the population. Furthermore, in recent decades, several anti-CVL vaccine candidates have been proposed [49]–[53]. In Brazil, two vaccines (Leish-Tec and Leishmune) [54]–[55] are commercially available, but they are not being used as control measures by the VLCSP. Recently, the LiESP/QA-21 vaccine (CaniLeish, Virbac, France) was launched in Europe [56]. Only 4.2% of the dogs in our study had been vaccinated against leishmaniasis, and thus the power was too low to detect association.

One limitation of cohort studies is the attrition rate due to loss of follow-up; however, a comparison of several features indicated that the dogs lost from the current study did not differ from those that remained in the study. Therefore, the effect of loss to follow-up was minimal. Another possible limitation of this study was the nonrandom selection of PCR-negative dogs from among those with negative screening test results. However, the selection of these dogs in proximity to PCR-positive dogs meant that all dogs were from similar environments. This study was designed not to evaluate a representative sample of Belo Horizonte but to assess the incidence of and identify risk factors for seroconversion. However, the northwest sanitary district is representative of the city as far as buildings, commerce, residences, and green areas.

Our results confirmed that a positive molecular test result preceded seroconversion. However, it is believed that seroconversion alone does not reflect the infectivity potential of the dogs and does not predict the progression of the infection to overt disease or to subpatent infection. It is important to mention that removing seropositive dogs without implementing environmental management measures does not prevent future infections in other animals [57].

In conclusion, domiciles that were most vulnerable to CVL occurrence were identified. Variables related to the backyard, such as the presence of leaves or manure, were maintained in the final model, as was insecticide spraying. Although short fur was an important risk factor for seroconversion that could be used to identify more-susceptible dogs, this characteristic cannot be modified and, consequently, is not a useful intervention target. Among the risk factors that can be controlled are: maintaining insecticide spraying in the house and health education focusing on cleaning backyards. Owners may also avoid clipping their dogs’ fur too short, keep their dogs indoors during periods of intense vector activity, and should maintain a clean yard without dry leaves or manure. The adoption of these measures can contribute to reduce the incidence rates. Furthermore, PCR-positive dogs should be monitored owing to the possibility of seroconversion, because control of HVL depends on the management of CVL.

## Supporting Information

Supporting Information S1 STROBE checklist.
(DOC)
